# Association between vitamin B6 status and liver fibrosis: evidence from NHANES 2005–2010

**DOI:** 10.3389/fnut.2025.1564257

**Published:** 2025-08-05

**Authors:** Junlu Peng, Wenjun Zhang, Meng Pan, Yuanlin Yu, Xiaopeng Chen

**Affiliations:** ^1^Department of Hepatobiliary Surgery, Shandong Provincial Third Hospital, Cheeloo College of Medicine, Shandong University, Jinan, China; ^2^Department of Hepatobiliary Surgery, Yijishan Hospital, Wannan Medical College, Wuhu, China; ^3^Department of Hepatobiliary Surgery, The Second Affiliated Hospital of Wannan Medical College, Wuhu, China

**Keywords:** vitamin B6, 4-pyridoxine acid, pyridoxal 5′-phosphate, metabolic rate, liver fibrosis

## Abstract

**Introduction:**

Liver fibrosis (LF) is a chronic liver disease with an increasing global prevalence, caused by various underlying factors. Vitamin B6 is significantly altered during inflammation and has been associated with diabetes and hypertension. To date, no study has explored the association between vitamin B6 and LF. We aimed to explore the association between vitamin B6 metabolites—4-pyridoxine acid (4-PA), pyridoxal 5′-phosphate (PLP), and the 4-PA/PLP ratio (an indicator of vitamin B6 metabolism rate in the body)—and LF risk.

**Methods:**

Data were extracted from the National Health and Nutrition Examination Surveys 2005–2010. Serum levels of 4-PA, PLP, and the 4-PA/PLP ratio were used to assess vitamin B6 status. The LF score was used to assess LF. Univariate and multivariate logistic regression tests were performed to explore the association between the vitamin B6 status and LF, using odds ratios (ORs) and 95% confidence intervals (CIs). Subgroup analysis based on age, obesity, and several complications was also conducted.

**Results:**

Of the 8,063 subjects, 741 (9.19%) had LF. After adjusting all covariates, we observed that a high PLP level was associated with a lower risk of liver fibrosis (OR = 0.44, 95% CI: 0.35–0.56, *p* < 0.001), while a high 4-PA/PLP ratio was associated with a higher risk of liver fibrosis (OR = 2.69, 95% CI: 1.87–3.86, *p* < 0.001). No significant association was found between 4-PA and LF. All of these associations remained robust in each subgroup based on age, obesity, as well as hypertension, hyperlipidemia, diabetes, and cardiovascular disease.

**Conclusion:**

In conclusion, a high serum PLP level was associated with lower odds of LF, whereas a high 4-PA/PLP ratio, representing an increased metabolic rate of vitamin B6, was associated with higher odds of LF.

## 1 Introduction

Liver fibrosis (LF) is a common chronic liver disease that commonly results from excessive deposition and abnormal reaction of extracellular matrix following hepatic injury, replacing normal liver tissue with fibrous tissue (1, 2). Its incidence rate has been increasing every year, becoming an important public health issue worldwide (2). If LF is chronic, it can develop into irreversible cirrhosis or even liver cancer (3). Clinical trials have shown that LF, as a precursor to cirrhosis and advanced cirrhosis—which is a significant risk factor for hepatocellular carcinoma—can be effectively alleviated and even reversed by eliminating the cause and providing appropriate treatment (4). Therefore, early diagnosis of LF is crucial for improving the treatment outcomes and prognosis of patients with liver disease.

Studies have shown that the pathogenesis of LF mainly involves multiple mechanisms, such as stem cell damage, inflammatory response, oxidative stress, collagen deposition, fibroblast activation, and extracellular matrix synthesis (1, 3). Vitamin B6 is a general term for a class of pyridoxines, mainly including pyridoxal, pyridoxine, and pyridoxamine, that are cofactors involved in various metabolic activities, such as amino acid, fat, and glucose metabolism (5, 6). Pyridoxal 5-phosphate (PLP), a phosphate derivative, is the biologically active form of this vitamin and demonstrates its long-term storage in the human body (7). In recent years, multiple studies have shown that vitamin B6 is associated with chronic stages of metabolic disorders such as diabetes (T2DM) and atherosclerosis. A small-scale, single-center, prospective study indicated that the deficiency rate of trace elements in patients with liver cirrhosis was high and that the deficiency rate of of vitamin B6 reached 60.8% (8). An animal study supported the ability of pyridoxamine to reduce LF (9). A multi-omics analysis revealed that vitamin B6 metabolites may play an important role in the pathogenesis of liver cirrhosis (10). The association between PLP and LF still needs to be further explored in large sample population studies.

Additionally, previous studies have demonstrated that vitamin B6 metabolism is significantly altered during inflammation, with PLP levels decreasing and 4-pyridoxine acid (4-PA), a catabolite of vitamin B6, increasing (11). Therefore, an indicator reflecting the metabolic rate of vitamin B6, the 4-PA/PLP ratio, was proposed and used to predict the prognosis of patients with T2DM and hypertension (12). Previous studies have suggested that a higher 4-PA/PLP ratio was positively associated with long-term outcomes in patients with T2DM or hypertension (12, 13). However, the relationship between 4-PA, PLP, and their ratio with the likelihood of LF is less understood.

Based on previous studies, we speculate that vitamin B6, as well as the rate of metabolism, are associated with the odds of LF. We anticipate a favorable correlation between vitamin B6 levels and metabolic rate with the likelihood of LF. This expectation is based on previous studies that have indicated that higher levels of vitamin B6 are correlated with improved metabolic functions, which may reduce the risk of LF (14). Herein, we tested this hypothesis using the National Health and Nutrition Examination Surveys (NHANES) database.

## 2 Methods

### 2.1 Study design and data sources

Using the NHANES database, we conducted this cross-sectional study to explore the association between vitamin B6 status and LF risk. NHANES is a cross-sectional study conducted by the Centers for Disease Control and Prevention (CDC) and the National Center for Health Statistics (NCHS) in the United States, using a complex, stratified, multistage sampling method to obtain a nationally representative sample. Since 1999, NHANES data have been released biennially and include participants’ demographic information, diet, examination, laboratory, questionnaire, and access-restricted data (available at: https://www.cdc.gov/nchs/nhanes/?CDC_AAref_Val=https://www.cdc.gov/nchs/nhanes/index.htm). All NHANES procedures are approved by the NCHS Research Ethics Review Board, and written informed consent was obtained from all participants.

### 2.2 Study population

**Figure 1:**
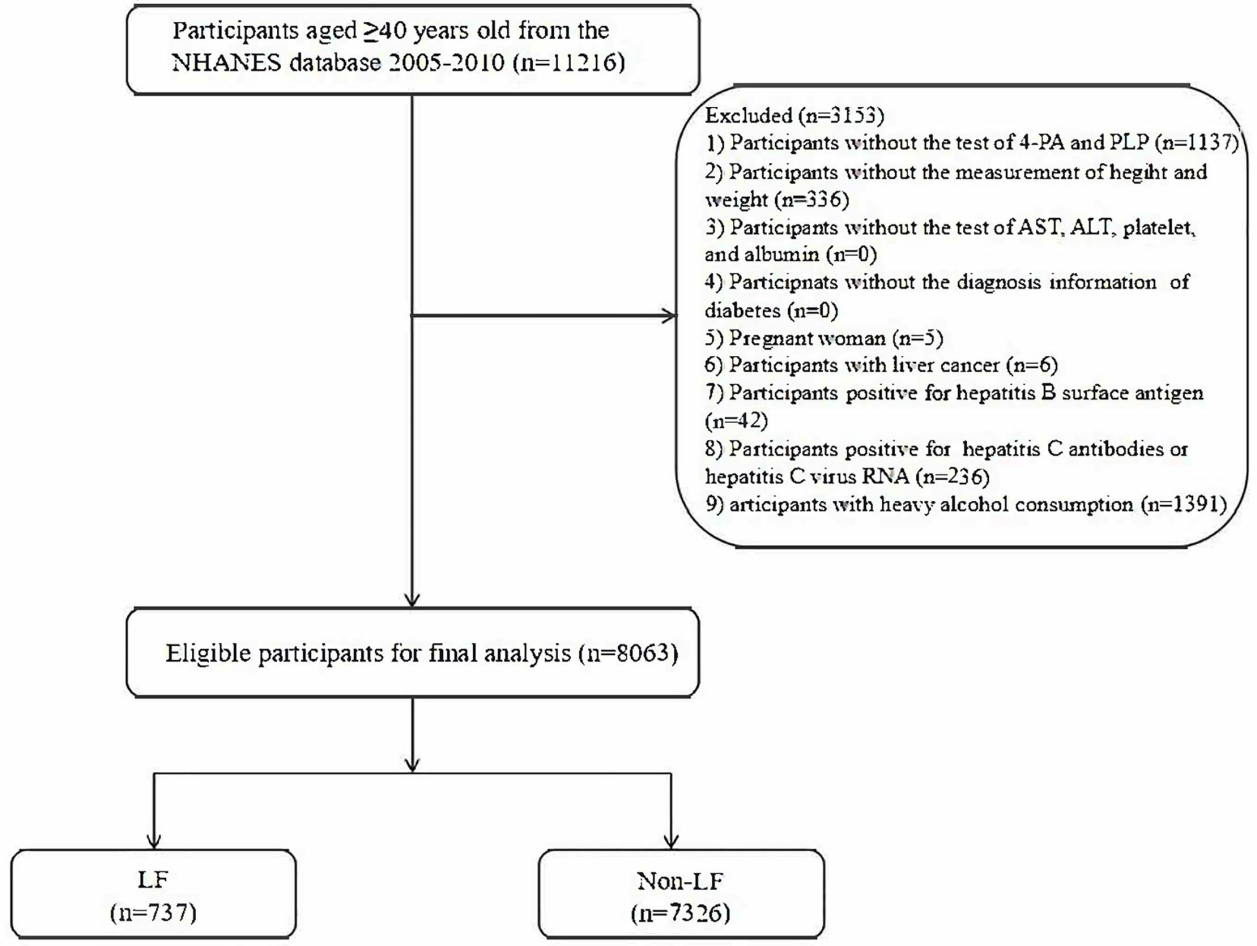
The flowchart of the screening of participants.

In total, 11,216 subjects aged 40 years and older were initially extracted from the NHANES 2005–2010 database. Then, 1,137 subjects without information regarding 4-PA or PLP, 336 subjects without information of height and weight, 5 pregnant women, 6 subjects with liver cancer history, 42 subjects with positive hepatitis B surface antigen, 236 subjects with positive hepatitis C surface antigen, and 1,391 subjects with heavy alcohol consumption were further excluded. We also considered the levels of aspartate aminotransferase (AST), alanine aminotransferase (ALT), platelets, albumin, history of T2DM, and fasting blood glucose during enrollment. We made sure that no information regarding the above laboratory indicators or disease history was missing. Finally, 8,063 eligible subjects were included for further analysis. Figure 1 shows the flowchart of the screening of participants.

### 2.3 Inclusion and exclusion criteria

#### 2.3.1 Inclusion criteria

The inclusion criteria for the participants were as follows: (1) age ≥ 40 years; (2) measured levels of PLP and 4-PA; and (3) available information on height, weight, glucose levels or T2DM status, AST, ALT, platelet count, and albumin levels for calculating the non-alcoholic fatty liver fibrosis score (NFS).

#### 2.3.2 Exclusion criteria

Participants were excluded if they were (1) pregnant, (2) had a history of liver cancer, (3) positive for hepatitis B surface antigen, (4) positive for hepatitis C antibody or hepatitis C virus RNA, or (5) heavy alcohol consumers.

### 2.4 Definition of LF

We used the validated non-invasive diagnostic index, the non-alcoholic fatty liver fibrosis score (NFS), to define LF. In the NAFLD cohort, NFS has been shown to have superior predictive performance (15, 16). LF was defined as NFS > 0.676 (17). The calculation of NFS is as follows:


$$\begin{array}{l} \mathrm{NFS}=[-1.675+0.037\times \mathrm{age}\;(\text{years})+0.094\times \mathrm{BMI}(\mathrm{kg}/m^{2})\;\\ +1.13\times \{\text{impaired fasting glycemia or}\;T2\mathrm{DM}\\ (\mathrm{yes}=1,\mathrm{no}=0)\}+0.99\times \mathrm{AST}/\mathrm{ALT}\;\text{ratio}-0.013\times \text{platelet}\\ (\times 10^{9}/L)-0.66\times \text{albumin}(g/\mathrm{dL})]. \end{array}$$

Subjects were assigned 1 point if their fasting blood glucose was ≥110 mg/dL, or if they had a history of T2DM, and 0 points otherwise.

### 2.5 Measurement of PLP, 4-PA

The present study utilized the levels of PLP (nmol/L) and 4-PA (nmol/L) and their ratio to assess vitamin B6 status. The detailed laboratory procedures for measuring the level of PLP and 4-PA are described in the NHANES documentation. High-performance liquid chromatography was used to determine the serum PLP and 4-PA levels. In the present study, 4-PA, PLP, and 4-PA/PLP ratios were categorized according to the tertiles of the study population (18, 19).

### 2.6 Potential covariates

We extracted the demographics [age, gender, race, poverty-to-income (PIR), and education level], lifestyle information [smoking, drinking, and physical activity], physical examination [height, weight, body mass index (BMI), diastolic blood pressure (DBP), and systolic blood pressure (SBP)], laboratory parameters [ALT, AST, platelet, albumin, glycated hemoglobin (HbA1c), fasting glucose, 2-h oral glucose tolerance test (OGTT), triglyceride (TG), total cholesterol (TC), high-density lipoprotein cholesterol (HDL-C), low-density lipoprotein cholesterol (LDL-C), c-reactive protein (CRP), and white blood cell (WBC) levels], systemic inflammation response index (SIRI), medication use (anti-platelet agent), and complications [T2DM, dyslipidemia, hypertension, and cardiovascular disease (CVD)].

PIR was divided into >1 (medium and high income) and ≤1 (poor or near poor). Participants who responded negatively to “Have you smoked at least 100 cigarettes in your entire life?” were considered never smokers; otherwise, they were denoted as smokers. Physical activity was expressed as a metabolic equivalent task (MET) and was calculated as follows: physical activity (met·min/week) = recommended MET × exercise time for corresponding activities (min/day) × the number of exercise days per week (day) (20). BMI (kg/m^2^) was the ratio of weight (kg) to the square of height (m) and divided into thin/normal (<25), overweight (25–29.9), and obese (≥30).

We define T2DM as meeting any of the following criteria: (1) HbA1c ≥ 6.5%, (2) fasting glucose ≥ 126 mg/dL, (3) 2-h OGTT ≥ 200 mg/dL, or (4) a self-report of clinician’s diagnosis. For hypertension, the definition included meeting any of the following conditions: (1) SBP ≥ 130 mmHg and/or DBP ≥ 80 mmHg, (2) a self-report of clinician’s diagnosis, or (3) a current prescription for any hypertension drug. The assessment of dyslipidemia was based on the following: (1) TC ≥ 200 mg/dL or TG ≥ 150 mg/dL, (2) LDL-C ≥ 130 mg/dL (3.4 mmol/L) or HDL-C ≤ 40 mg/dL (1.0 mmol/L), (3) a self-report of doctors’ diagnosis, or (4) a current prescription for dyslipidemia. CVD was defined by the question, “Have you ever been told that you had angina/heart attack/coronary heart disease/stroke/congestive heart failure?” SIRI was calculated according to the formula (21): SIRI = (monocyte count × neutrophil count)/lymphocyte count.

### 2.7 Statistical analysis

Data analysis was conducted using the R project (version 4.2.3). Continuous data were represented as mean ± standard deviation (SD), and a *t*-test was performed to compare the LF and non-LF groups. Categorical data were represented as numbers and percentages, and a chi-squared test was used to compare the two groups. Covariates were selected based on a hybrid approach that integrated prior knowledge and data-driven screening. First, clinically established confounders (e.g., age, sex, and ethnicity) were included *a priori*, regardless of statistical significance. Second, candidate variables were screened using univariate regression (18). Missing data were imputed using multiple imputation based on random forest. Sensitivity analysis was also performed on the data before and after imputation to verify its robustness (Supplementary Table 1). The univariate logistic analysis was used to identify the covariates related to LF (Supplementary Table 2). The multivariate logistic models were constructed to explore the association between 4-PA, PLP, and 4-PA/PLP ratios and the odds of LF, using the values of odds ratio (OR) and 95% confidence interval (CI). Subgroup analysis, based on age, obesity, hypertension, hyperlipidemia, T2DM, and CVD, was performed to further verify whether the association between LF and 4-PA, PLP, and 4-PA/PLP ratios remains robust. A *p*-value of <0.05 was considered significant.

## 3 Results

### 3.1 Characteristics of the population

We recruited 8,063 subjects from the NHANES database 2005–2010, with a mean age of 57.43 (±0.30) years. Among them, 741 (9.19%) had LF. Table 1 shows the characteristics of the study population. The percentage of participants with higher 4-PA/PLP (≥0.939) was significantly higher in the LF group than in the non-LF group (62.85% *vs.* 31.34%). Significant differences were found between age, ethnicity, PIR level, education, physical activity, BMI, energy intake, CRP, SIRI, the history of T2DM, dyslipidemia, hypertension, CVD, and the use of an anti-platelet agent (all *p* < 0.05).

**Table 1:** Characteristics of study population.

Variables	Total (*N* = 8,063)	Non-LF (*N* = 7,322)	LF (*N* = 741)	*P*
4-PA, nmol/L, Mean (±S E)	126.64 (±9.42)	126.02 (±9.58)	135.91 (±22.10)	0.652
4-PA, nmol/L, n (%)				0.003
<22.4	2,978 (33.22)	2,761 (33.68)	217 (26.27)	
≥44.9	2,703 (36.92)	2,404 (36.50)	299 (43.07)	
22.4–44.9	2,382 (29.87)	2,157 (29.81)	225 (30.67)	
PLP, nmol/L, Mean (±S.E)	77.95 (±1.74)	79.56 (±1.79)	54.01 (±2.32)	<0.001
PLP, nmol/L, *n* (%)				<0.001
<33.1	3,033 (33.25)	2,645 (32.08)	388 (50.74)	
≥70.3	2,359 (33.40)	2,215 (34.34)	144 (19.38)	
33.1–70.3	2,671 (33.35)	2,462 (33.58)	209 (29.87)	
4-PA/PLP ratio, Mean (±S.E)	1.17 (±0.03)	1.11 (±0.03)	2.11 (±0.26)	<0.001
4-PA/PLP ratio, *n* (%)				<0.001
<0.571	2,694 (33.33)	2,606 (34.95)	88 (9.24)	
≥0.939	2,832 (33.32)	2,378 (31.34)	454 (62.85)	
0.571–0.939	2,537 (33.35)	2,338 (33.71)	199 (27.91)	
Age, years, Mean (±S.E)	57.43 (±0.30)	56.58 (±0.29)	70.08 (±0.57)	<0.001
Gender, n (%)				0.503
Female	4,500 (57.57)	4,107 (57.68)	393 (55.95)	
Male	3,563 (42.43)	3,215 (42.32)	348 (44.05)	
Race, *n* (%)				<0.001
Mexican American	1,295 (5.63)	1,206 (5.71)	89 (4.41)	
Non-Hispanic Black	1,492 (9.50)	1,320 (9.18)	172 (14.24)	
Non-Hispanic White	4,265 (75.83)	3,859 (75.87)	406 (75.26)	
Other Race	1,011 (9.04)	937 (9.23)	74 (6.09)	
PIR, *n* (%)				0.014
>1	6,226 (85.26)	5,678 (85.54)	548 (81.05)	
≤1	1,150 (8.17)	1,034 (7.99)	116 (10.84)	
*U*nknown	687 (6.57)	610 (6.47)	77 (8.11)	
Education, n (%)				<0.001
Above high school	5,711 (82.24)	5,270 (83.15)	441 (68.68)	
Below high school	1,187 (7.07)	1,031 (6.55)	156 (14.85)	
High school/GED or equivalent	1,165 (10.69)	1,021 (10.31)	144 (16.47)	
PA, n (%)				<0.001
<750	5,061 (58.72)	4,511 (58.00)	550 (69.58)	
≥750	2093 (30.13)	1983 (30.90)	110 (18.62)	
Unknown	909 (11.15)	828 (11.11)	81 (11.80)	
Smoking, *n* (%)				0.777
No	4,432 (56.09)	4,033 (56.14)	399 (55.36)	
Yes	3,631 (43.91)	3,289 (43.86)	342 (44.64)	
BMI, *n* (%)				<0.001
Underweight/Normal weight	2050 (28.13)	2005 (29.64)	45 (5.61)	
Overweight	2,889 (35.02)	2,700 (35.70)	189 (24.86)	
Obesity	3,124 (36.85)	2,617 (34.65)	507 (69.53)	
VB6 intake, mg, Mean (±S.E)	4.72 (±0.23)	4.77 (±0.26)	3.95 (±0.58)	0.245
Energy intake, kcal, Mean (±S.E)	2026.29 (±17.82)	2043.48 (±17.96)	1769.91 (±52.64)	<0.001
CRP, mg/dL, Mean (±S.E)	0.42 (±0.01)	0.40 (±0.01)	0.62 (±0.04)	<0.001
SIRI, 1000cells/μl, Mean (±S.E)	1.24 (±0.02)	1.23 (±0.02)	1.43 (±0.05)	<0.001
Diabetes, *n* (%)				<0.001
No	6,136 (81.87)	5,919 (85.36)	217 (29.70)	
Yes	1927 (18.13)	1,403 (14.64)	524 (70.30)	
Dyslipidemia, *n* (%)				0.014
No	1,650 (20.75)	1,527 (21.04)	123 (16.44)	
Yes	6,413 (79.25)	5,795 (78.96)	618 (83.56)	
Hypertension, *n* (%)				<0.001
No	2,311 (31.01)	2,209 (32.12)	102 (14.48)	
Yes	5,752 (68.99)	5,113 (67.88)	639 (85.52)	
CVD, *n* (%)				<0.001
No	6,869 (88.34)	6,400 (90.04)	469 (62.89)	
Yes	1,194 (11.66)	922 (9.96)	272 (37.11)	
Anti-platelet agent, *n* (%)				<0.001
No	7,789 (97.34)	7,114 (97.76)	675 (91.04)	
Yes	274 (2.66)	208 (2.24)	66 (8.96)	

LF, liver fibrosis; 4-PA, 4-pyridoxic acid; PLP, pyridoxal 5′-phosphate; PIR, poverty-to-income ratio; PA, physical activity; BMI, body mass index; VB6, vitamin B6; CRP, c-reactive protein; SIRI, systemic inflammatory response index; CVD, cardiovascular disease.

### 3.2 Association between 4-PA, PLP, and 4-PA/PLP ratios and LF

We conducted two logistic regression models to explore the association between 4-PA, PLP, and 4-PA/PLP ratios with the risk of liver fibrosis, which is depicted in Table 2. After adjusting for the covariates, including age, ethnicity, PIR, education, physical activity, BMI, CRP, SIRI, hypertension, dyslipidemia, CVD, and an anti-platelet agent, we found that higher levels of PLP were associated with the lower risk of LF (OR = 0.44, 95% CI: 0.35–0.56), and higher 4-PA/PLP ratio (≥0.939) was associated with the higher risk of LF (OR = 2.69, 95% CI: 1.87–3.86).

**Table 2:** Association between 4-PA, PLP and 4-PA/PLP ratio with the risk of LF.

Variables	Model 1		Model 2	
	OR (95%CI)	*P*	OR (95%CI)	*P*
4-PA, *n* (%)				
<22.4	Ref		Ref	
22.4–44.9	1.32 (1.02–1.71)	0.036	0.90 (0.66–1.23)	0.496
≥44.9	1.51 (1.27–1.81)	<0.001	0.86 (0.67–1.10)	0.227
PLP, *n* (%)				
<33.1	Ref		Ref	
33.1–70.3	0.56 (0.45–0.71)	<0.001	0.70 (0.53–0.94)	0.020
≥70.3	0.36 (0.29–0.43)	<0.001	0.44 (0.35–0.56)	<0.001
4-PA/PLP ratio, *n* (%)				
<0.571	Ref		Ref	
0.571–0.939	3.13 (2.14–4.58)	<0.001	2.07 (1.36–3.15)	0.001
≥0.939	7.58 (5.58–10.31)	<0.001	2.69 (1.87–3.86)	<0.001

LF, liver fibrosis; 4-PA, 4-pyridoxic acid; PLP, pyridoxal 5′-phosphate; Ref, reference; OR, odd ratio; CI, confidence interval; Model 1, crude model; Model 2, adjusted age, race, PIR, education, PA, BMI, energy intake, CRP, SIRI, hypertension, dyslipidemia, CVD and anti-platelet agent.

### 3.3 Association between 4-PA, PLP, and 4-PA/PLP ratios and LF based on age, obesity, and comorbidities

Subgroup analysis based on age, obesity, hypertension, hyperlipidemia, T2DM, and CVD was further conducted to verify the associations between 4-PA, PLP, and 4-PA/PLP ratios and LF risk. In a fully adjusted model, we found that the association of 4-PA, PLP, and 4-PA/PLP ratios with the risk of liver fibrosis remains robust in each subgroup. The results are shown in Supplementary Figures.

## 4 Discussion

In the present cross-sectional study, we selected 8,063 subjects from the NHANES database between 2005 and 2010 who had LF assessment information and vitamin B6 levels to explore the association between vitamin B6 status and LF. After adjusting for all covariates affecting LF, we observed that high PLP level was associated with a lower risk of LF, while high 4-PA-to-PLP ratio was associated with a higher LF risk. No significant association between 4-PA and LF was observed. All these associations remain robust in each subgroup.

In recent years, several epidemiological and animal studies have reported the association between vitamin B6 and metabolic chronic diseases. Nakamura et al. (22) attempted to utilize the rates of streptozocin-induced diabetic nephropathy to elucidate whether PLP had any beneficial effects on diabetic nephropathy and reported desirable results demonstrating that PLP could prevent the progression of diabetic nephropathy in rats by inhibiting the formation of advanced glycation end-products. A cohort from Germany also reported that vitamin B6 deficiency is a common phenomenon in patients with T2DM, and early kidney disease is significantly associated with changes in vitamin B6 metabolism (23). A Framingham Heart Study cohort reported that a low PLP level is independently associated with higher CRP. This observation may suggest that patients with CVD and a persistent inflammatory state may benefit from higher vitamin B6 levels (24). Moreover, the role of vitamin B6 in immunity has also been widely discussed. Vitamin B6 can promote the proliferation and differentiation of lymphocytes and improve their ability to recognize and attack pathogens. In animal studies, mice deficient in vitamin B6 exhibited reduced lymphocyte proliferation, which affected T-cell-mediated cytotoxicity, cytokine and chemokine production, delayed hypersensitivity reactions, and changes in allogeneic transplantation responses. Vitamin B6 supplementation was therefore demonstrated to effectively improve immune responses (5). Reduced intake of vitamin B6 has been established as a cancer risk factor. The incidence of diseases, such as pancreatic cancer (25), gastric adenocarcinoma (26), colorectal cancer (27), and lung cancer (28), showed a significant negative correlation with vitamin B6 or serum PLP levels. Recent research indicating similar pathways of fibrosis development in humans has been incorporated into our study, supported by clinical data associating vitamin B6 levels with liver function. This enhancement clarifies the relevance of our findings and provides a more robust connection between animal studies and human LF (27).

Based on existing research, the association between elevated PLP levels and reduced LF risk may involve the following mechanisms:

(1) Antioxidant Stress Regulation: As an active form of vitamin B6, PLP regulates glutathione metabolism-related proteins. Studies demonstrate that PLP upregulates glutathione S-transferase alpha-4 (GSTA4) expression, enhancing hepatic defense against oxidative damage, and thereby inhibiting hepatic stellate cell (HSC) activation and collagen deposition. (2) Transaminase Activity Normalization: In chronic liver disease, PLP deficiency—as a coenzyme of AST/ALT—may cause pseudonormalization of transaminase activity, compromising the diagnostic accuracy of the AST/ALT ratio. Guéchot et al. (29) confirmed that non-standardized aminotransferase measurements invalidate AST/ALT as indicators of fibrosis. PLP supplementation restores accurate transaminase quantification, reflecting the true extent of liver injury and preventing the underestimation of fibrosis. (3) Metabolic Pathway Modulation: Proteomic analyses revealed that PLP regulates the branched-chain amino acid and cysteine metabolism pathways. This reduces toxic metabolite accumulation and improves the hepatic microenvironment, suppressing fibrogenesis—consistent with findings where polysaccharide extracts attenuated fibrosis through similar metabolic reprogramming (30). (4) Key Signaling Pathway Inhibition: Natural compounds (e.g., rutin) exert antifibrotic effects by inhibiting mTOR/p70 S6K pathways. PLP likely modulates HSC proliferation/activation through analogous mechanisms, as predicted through *in silico* screening by Thiyagarajan et al. (31). Caution: High-dose PLP (e.g., 100 mg/kg/day) may induce hepatotoxicity (32), suggesting dose-dependent protective effects. Thus, maintaining physiological PLP levels reduces LF risk through multiple mechanisms but requires avoidance of excessive intake.

### 4.1 Mechanisms linking the elevated 4-PA/PLP ratio with increased LF risk

Current evidence suggests that the elevated 4-PA/PLP ratio promotes LF through the following mechanisms.

#### 4.1.1 Vitamin B6 metabolic imbalance

Elevation of 4-PA—the terminal oxidation product of PLP—reflects in an excessive conversion of active PLP to inactive forms. Resultant PLP deficiency impairs transaminase activity (e.g., AST/ALT), masking true liver injury severity and delaying LF diagnosis (29).

#### 4.1.2 Exacerbated oxidative stress

PLP deficiency reduces GSTA4-mediated antioxidant capacity, diminishing free radical clearance and promoting HSC activation/collagen deposition.

#### 4.1.3 Toxic metabolite accumulation

Impaired PLP-dependent metabolism of branched-chain amino acids (e.g., BCKDHA regulation) and cysteine elevates hepatotoxic intermediates (e.g., homocysteine), thereby directly damaging hepatocytes and activating profibrotic pathways.

#### 4.1.4 Drug metabolism abnormalities

Chronic high-dose PLP therapy (e.g., in PNPO deficiency) may induce direct hepatotoxicity via PLP or its metabolites (e.g., 4-PA). Sudarsanam et al. (32) documented cirrhosis in patients receiving PLP replacement, while animal models showed abnormal pyridoxal/pyridoxic acid elevation, suggesting early cirrhosis.

Elevated 4-PA/PLP ratio signifies dysregulated vitamin B6 metabolism or PLP utilization defects, exacerbating hepatic injury and LF through multiple mechanisms. Although maintaining a physiological PLP balance has hepatoprotective benefits, it necessitates caution against over-supplementation toxicity. A recent study by Zhang et al. (33) further emphasizes the importance of phospholipid signaling (e.g., Plpp3) in fibrosis modulation.

To date, very few population-based epidemiological studies have been performed on the association between vitamin B6 and LF. The results of this study are consistent with and extend previous observations. The association between PLP and 4-PA/PLP ratios and LF persisted after adjusting for several confounders, including demographics, comorbidities, lifestyle, and treatment. Moreover, these findings were corroborated by subgroup analysis, indicating that the association between vitamin B6 status and LF was robust across populations with different characteristics. Several potential biological mechanisms could support the association between vitamin B6 and LF. LF refers to the liver’s self-repair and wound-healing processes, which are activated by external events, resulting from a complex multicellular response. Although LF has different pathogenic factors, its formation process is very similar, including long-term chronic parenchymal damage, continuous activation of inflammatory response and oxidative stress, massive deposition of extracellular matrix, and fibrous scar formation, which together destroy the normal structure and function of the liver (1, 3). The anti-inflammatory activity is one of the most comprehensive biological roles of vitamin B6. In lipopolysaccharide-induced macrophages, vitamin B6 can reduce the mRNA and protein expression levels of nitric oxide synthase and cyclooxygenase-2 by inhibiting the activation of the nuclear factor-kB signaling pathway (34). In addition, vitamin B6 can also interfere with the activation of the NOD-like receptor protein 3 signaling pathway, thereby inhibiting the maturation and secretion of interleukin-1β and interleukin-18 (35). It can also reduce superoxide free radical content and lipid peroxidation levels in H_2_O_2_-treated vascular endothelial cells. Vitamin B6 functions as a coenzyme in the glutathione antioxidant defense system and is also a crucial component in the supersulfur reaction, facilitating the conversion of homocysteine into cysteine, which is a necessary substrate for glutathione production (36). Therefore, vitamin B6 may directly or indirectly participate in antioxidant defense. Although we listed some possible biological mechanisms of vitamin B6 in LF, the potential biological mechanisms involving the two still need to be further studied.

### 4.2 Research strengths and clinical implications

This study provides the first investigation into the association of vitamin B6 status and its catabolic profile (4-PA, PLP, and 4-PA/PLP ratios) with LF, offering valuable insights for fibrosis prevention strategies. Our study has several advantages. First, our study was based on the NHANES, a large, public, and well-representative database, where 8,063 participants with complete clinical data required for the study to explore the association of vitamin B6 levels and its metabolites with LF were included, which made our results more representative. Second, considering that the epidemiological attributes of LF are influenced by numerous factors, such as demographic characteristics and health status, we accounted for the confounding variables, including age, race, PIR, educational attainment, physical activity, BMI, energy consumption, CRP, SIRI, hypertension, dyslipidemia, CVD, and anti-platelet medication, to enhance the precision of the analytical outcomes. Certainly, other medications that may influence the results have not been extensively described here. These variables will be included in subsequent studies for further analysis (14). In addition, we further implemented subgroup analyses based on age, BMI, hypertension, hyperlipidemia, T2DM, and CVD. All subgroup analysis results showed that PLP and 4-PA/PLP ratios were associated with the risk of LF. Third, we used NFS to assess LF. Previous studies have demonstrated that NFS has high accuracy in predicting LF and can predict liver disease-related prognosis (37). NFS is user-friendly and practical, and it is rational and methodical to stratify patients with LF based on risk. Fourth, our study suggests that simple 4-PA/PLP measurement in plasma may provide more valuable risk stratification information than traditional LF risk factors, thereby guiding clinicians to implement targeted intervention measures.

## 5 Limitations

Our study has several limitations that should be mentioned. First, due to the nature of the cross-sectional study, our study only supports the association of PLP and 4-PA/PLP ratio with the risk of liver fibrosis, but not a causal relationship. Second, we only had a single baseline measurement of the 4-PA/PLP ratio, which may not capture the trajectory of the changes in its level. The association between the dynamic changes in vitamin B6 status and LF still needs further research and analysis. Third, despite our consideration of several confounding factors associated with LF, including demographic data, comorbidities, and lifestyle behaviors, we cannot eliminate the possibility of residual confounding affecting the relationship between exposure and outcome. The association between vitamin B6 status in the human body and LF still needs to be further analyzed in future large-sample, well-designed, prospective cohort studies.

## 6 Conclusion

In summary, we found that a higher vitamin B6 metabolite rate, representing a low vitamin B6 level in the body, was positively associated with LF. Our findings supported the prospective investigation of the therapeutic significance of the 4-PA/PLP ratio as a straightforward risk marker to improve risk classification and facilitate targeted therapies in patients with LF.

## Data Availability

The original contributions presented in the study are included in the article/Supplementary material; further inquiries can be directed to the corresponding author.
