# Supplementary material for: Association between vitamin B6 status and liver fibrosis: evidence from NHANES 2005–2010
Source: Front Nutr. 2025 Aug 5;12:1564257. doi: 10.3389/fnut.2025.1564257 (PMC12360946; doi:10.3389/fnut.2025.1564257)
Supplement: Supplementary file 1 [file Table_1.docx]

Supplementary table 1 Sensitivity analyses before and after imputation of missing data

| Variables | Before imputation | After imputation | *P* |
| --- | --- | --- | --- |
| Dyslipidemia, n (%) |  |  | 0.325 |
| No | 1650 (20.75) | 1650 (20.75) |  |
| Yes | 6412 (79.25) | 6413 (79.25) |  |
| CRP, mg/dL, Mean (±S.E) | 0.42 (±0.01) | 0.42 (±0.01) | 0.359 |
| Smoking, n (%) |  |  | 0.652 |
| No | 4429 (56.09) | 4432 (56.09) |  |
| Yes | 3629 (43.91) | 3631 (43.91) |  |
| Energy, kcal, Mean (±S.E) | 2028.65 (±18.32) | 2026.29 (±17.82) | 0.069 |
| VB6 intake, mg, Mean (±S.E) | 4.74 (±0.24) | 4.72 (±0.23) | 0.150 |

CRP, c-reaction protein; VB6, vitamin B6.

Supplementary table 2 Univariate analysis of the risk of LF

| Variables | Outcome/Total | OR (95% CI) | *P* |
| --- | --- | --- | --- |
| Age, years, | N=741/8063 | 1.10 (1.09-1.11) | <0.001 |
| Gender |  |  |  |
| Female | N=393/4500 | Ref |  |
| Male | N=348/3563 | 1.07 (0.87-1.32) | 0.503 |
| Race |  |  |  |
| Mexican American | N=89/1295 | Ref |  |
| Non-Hispanic Black | N=172/1492 | 2.01 (1.50-2.69) | <0.001 |
| Non-Hispanic White | N=406/4265 | 1.29 (1.02-1.62) | 0.035 |
| Other Race | N=74/1011 | 0.86 (0.56-1.31) | 0.468 |
| PIR |  |  |  |
| >1 | N=548/6226 | Ref |  |
| ≤1 | N=116/1150 | 1.43 (1.12-1.83) | 0.005 |
| Unknown | N=77/687 | 1.32 (0.93-1.88) | 0.112 |
| Education |  |  |  |
| Above High School | N=441/5711 | Ref |  |
| Below High School | N=156/1187 | 2.75 (2.07-3.66) | <0.001 |
| High School/GED or Equivale | N=144/1165 | 1.93 (1.47-2.55) | <0.001 |
| PA |  |  |  |
| <750 | N=550/5061 | Ref |  |
| ≥750 | N=110/2093 | 0.50 (0.37-0.67) | <0.001 |
| Unknown | N=81/909 | 0.89 (0.63-1.25) | 0.479 |
| Smoking |  |  |  |
| No | N=399/4432 | Ref |  |
| Yes | N=342/3631 | 1.03 (0.83-1.29) | 0.777 |
| BMI |  |  |  |
| Underweight/Normal weight | N=45/2050 | Ref |  |
| Overweight | N=189/2889 | 3.68 (2.64-5.14) | <0.001 |
| Obesity | N=507/3124 | 10.61 (7.43-15.14) | <0.001 |
| VB6 intake, mg | N=741/8063 | 0.99 (0.98-1.01) | 0.355 |
| Energy intake, kcal | N=741/8063 | 1.00 (1.00-1.00) | <0.001 |
| CRP, mg/dL | N=741/8063 | 1.22 (1.12-1.33) | <0.001 |
| SIRI, 1000cells/μl | N=741/8063 | 1.25 (1.15-1.36) | <0.001 |
| Dyslipidemia |  |  |  |
| No | N=123/1650 | Ref |  |
| Yes | N=618/6413 | 1.35 (1.07-1.72) | 0.014 |
| Hypertension |  |  |  |
| No | N=102/2311 | Ref |  |
| Yes | N=639/5752 | 2.80 (2.12-3.69) | <0.001 |
| CVD |  |  |  |
| No | N=469/6869 | Ref |  |
| Yes | N=272/1194 | 5.34 (4.27-6.67) | <0.001 |
| Anti-platelet agent |  |  |  |
| No | N=675/7789 | Ref |  |
| Yes | N=66/274 | 4.31 (2.93-6.32) | <0.001 |

LF, liver fibrosis; PIR, poverty-to-income ratio; PA, physical activity; BMI, body mass index; VB6, vitamin B6; CRP, c-reaction protein; SIRI, systemic inflammatory response index; CVD, cardiovascular disease.
