# Supplementary figures and images for: Association between vitamin B6 status and liver fibrosis: evidence from NHANES 2005–2010
Source: Front Nutr. 2025 Aug 5;12:1564257. doi: 10.3389/fnut.2025.1564257 (PMC12360946; doi:10.3389/fnut.2025.1564257)

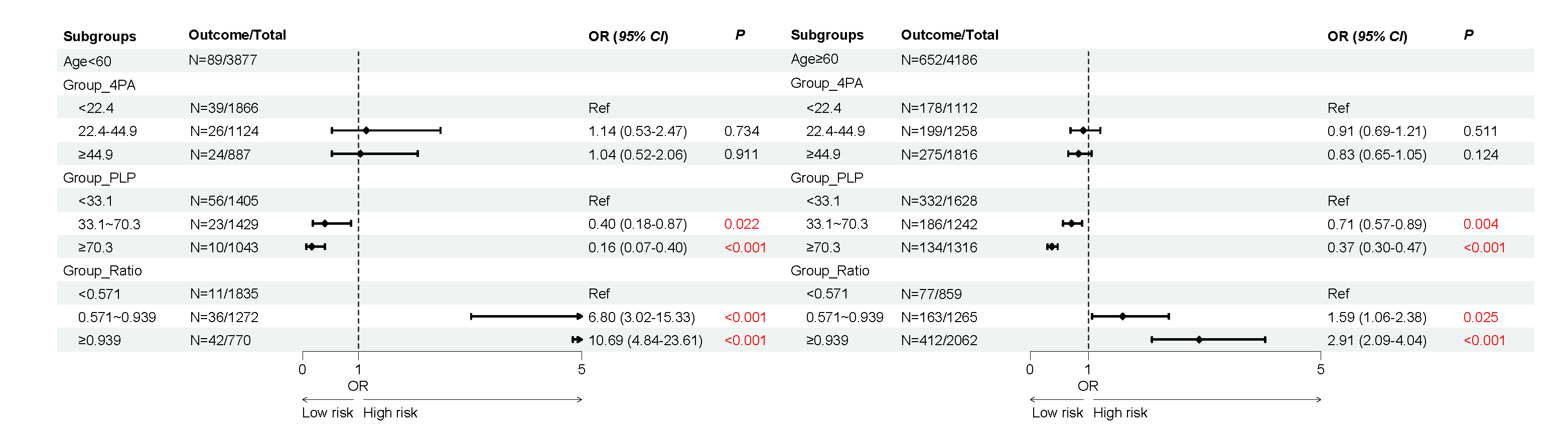

Supplement: Supplementary file 2 [file Image_1.tiff]

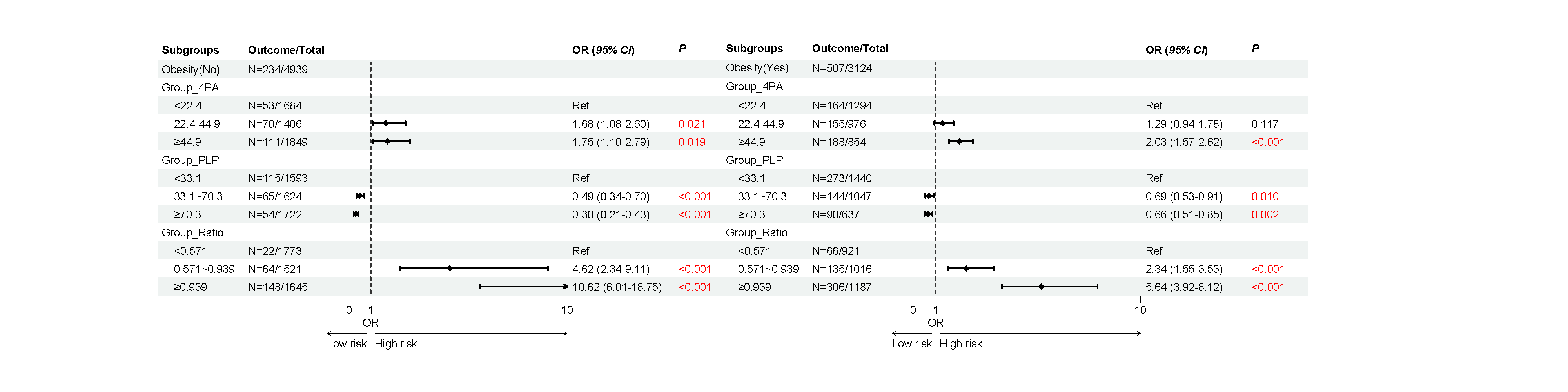

Supplement: Supplementary file 3 [file Image_2.tiff]

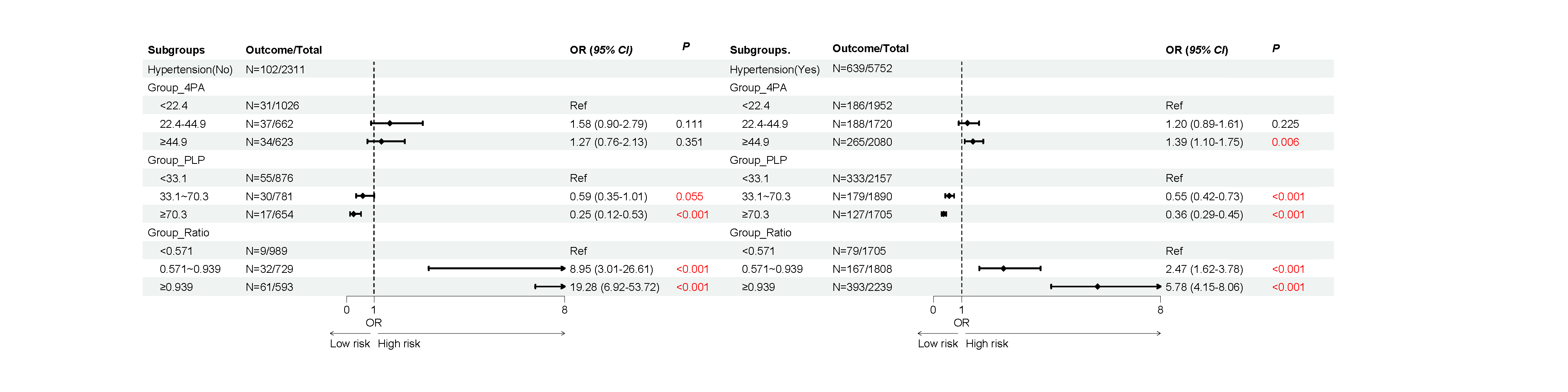

Supplement: Supplementary file 4 [file Image_3.tiff]

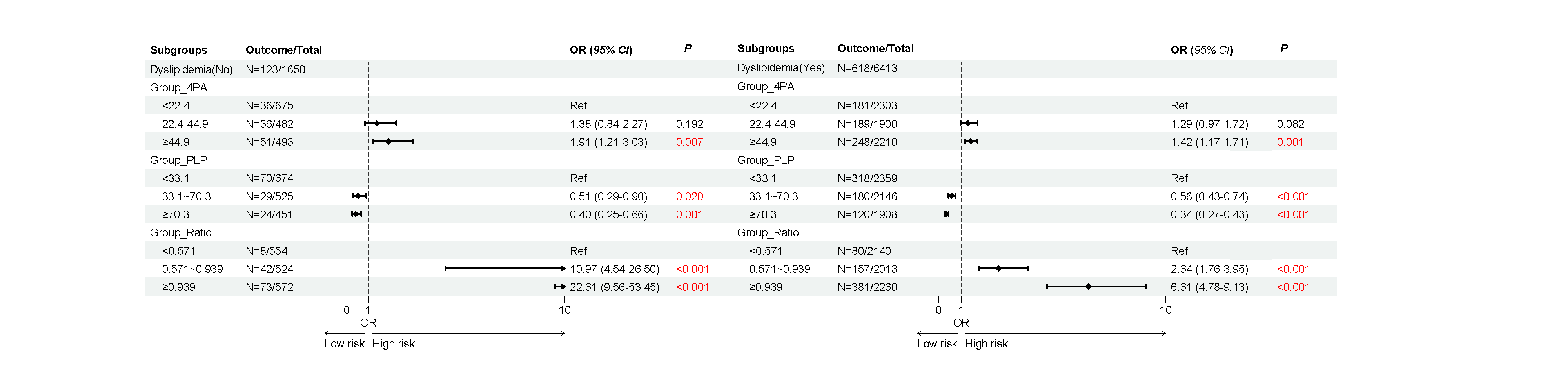

Supplement: Supplementary file 5 [file Image_4.tiff]

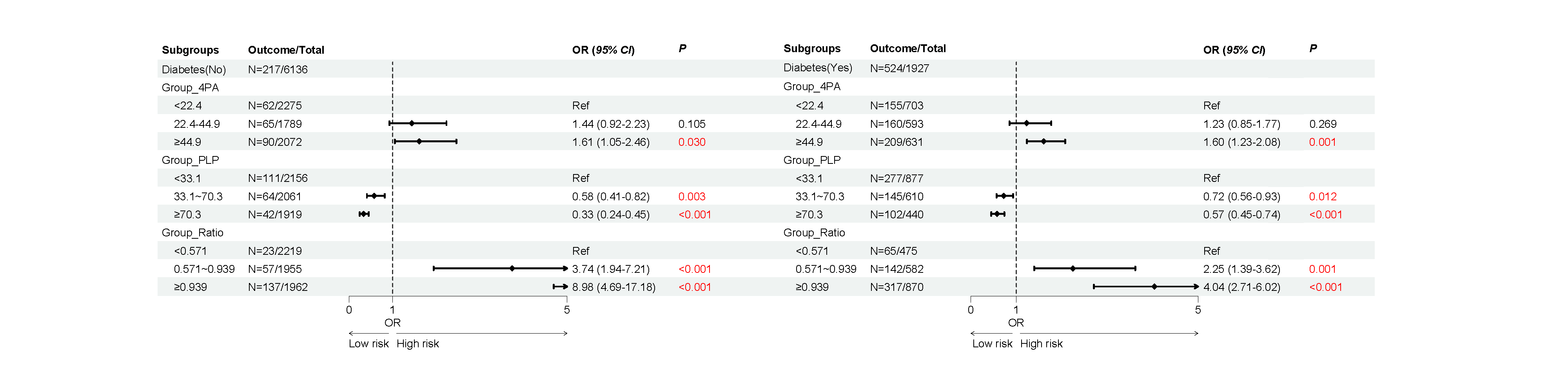

Supplement: Supplementary file 6 [file Image_5.tiff]

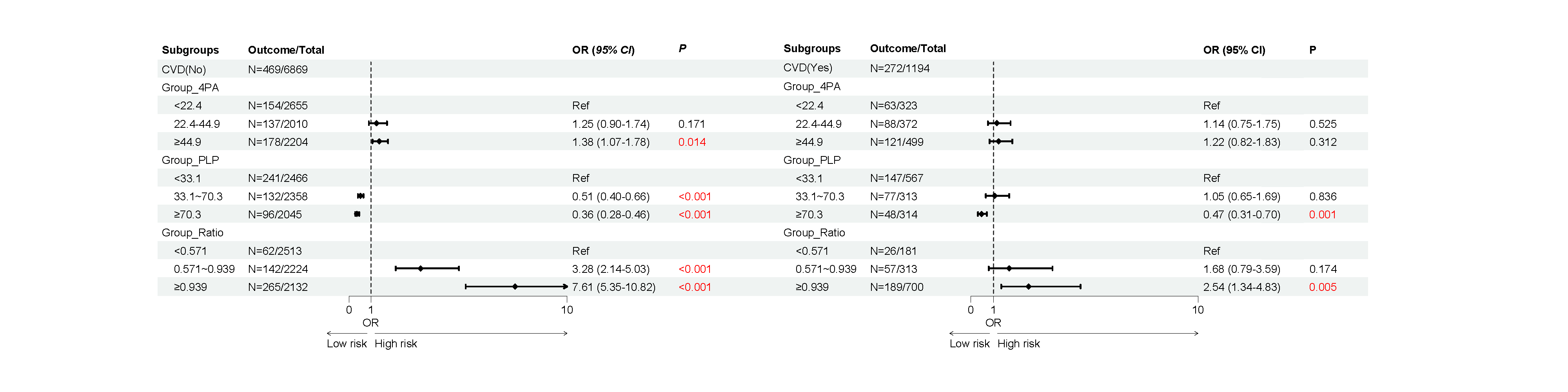

Supplement: Supplementary file 7 [file Image_6.tiff]
